# Acellular porcine Achilles tendon patch encapsulating tendon-derived stem cells for rotator cuff repair in a rabbit model

**DOI:** 10.1038/s41598-024-57495-z

**Published:** 2024-03-27

**Authors:** Huawei Wen, Yushun Fang

**Affiliations:** 1 Wuhan Fourth Hospital, Wuhan, 430030 China; 2 Wuhan Sports Medicine Center, Wuhan, 430030 China

**Keywords:** Acellular porcine Achill tendon patch, Tendon-derived stem cells, Rotator cuff repair, Enthesis, Stem-cell differentiation, Biomaterials, Structural materials, Tendons

## Abstract

Currently, the predominant method for repairing rotator cuff involves surgical suture techniques, but the failure rate remains notably high. Failure of the rotator cuff insertion to provide adequate biomechanics during early healing is considered a major cause of failure. Addressing this problem, biological augmentation emerges as a promising strategy for enhancing the biomechanical properties during early stages. Tendon-derived stem cells (TDSCs), which facilitate the differentiation of repair-supportive cells, hold the potential to improve the efficacy of patch application. The study aims to assess the behavior of TDSCs in acellular porcine Achilles tendon (APAT) patches and to explore the capacity of the APAT patch encapsulating TDSCs in promoting both tendon-to-bone healing and biomechanical enhancements in a rabbit rotator cuff repair model. Transmission electron microscopy (TEM) analyses validated the complete cellular clearance of native cells from APAT patches, with uniform distribution of TDSCs. Immunofluorescence staining confirmed successful TDSCs attachment, while population doubling time (PDT) underscored increased TDSCs proliferation on APAT patches. Quantitative polymerase chain reaction (qPCR) demonstrated upregulation of tenocyte and osteocyte related genes in TDSCS cultured within the patches. In the subsequent in vivo experiment, fifty-four rabbits were used to create rotator cuff injury models and randomly assigned to a control group, an APAT patch group, and an APAT patch with TDSCs group. Histological analysis showed that the APAT patch with TDSCs group had significantly enhanced tendon-to-bone healing and a distinctly organized tendon-fibrocartilage-bone structure, as compared to the APAT patch group. In addition, the biomechanical properties of the APAT patch with TDSCs group were significantly improved. In conclusion, APAT patches promote TDSC proliferation and stimulate tenogenic and osteogenic differentiation. APAT patches encapsulating TDSCs have shown considerable potential in promoting tendon-to-bone healing of rotator cuff injuries, indicating that their use in rotator cuff repair surgery is clinically meaningful.

## Introduction

Rotator cuff tears are common shoulder injuries that usually lead to disability, pain and a decline in quality of life. Within the United States, the annual count of rotator cuff repair operations exceeds 75,000, and a substantial proportion of repaired rotator cuff exhibit a pronounced failure rate, necessitating subsequent, financially burdensome revision surgeries, which contribute not only to heightened physical distress but also emotional duress^1,2^. Moreover, the number of degenerative rotator cuff tears is expected to increase with the aging of the active population. Currently, the predominant approach to addressing rotator cuff tears entails surgical suturing techniques that anchor the torn tendon to its point of insertion (enthesis) on the greater tuberosity of humerus. This enthesis can be categorized into approximately four transitional zones: tendon, uncalcified fibrocartilage, calcified fibrocartilage, and bone. However, transitions between zones are gradual and continuous. Furthermore, even at the ultrastructural level, there are no clearly-defined boundaries between the zones^3^. This unique structure functionally distributes the mechanical stress and enhances the bonding strength between the soft tendon and hard bone^4^. At present, the majority of research efforts are improving rotator cuff repair by promoting tendon-to-bone healing. Specifically, high initial fixation strength, mechanical stability, and biological healing of the enthesis are the main goals of the repair surgery. Therefore, biological augmentation approaches have been proposed to improve patient outcomes.

Biological patches may offer an effective solution for the treatment of rotator cuff tears, particularly, the massive and irreparable rotator cuff tears^5^. Various types of biological patches made of the tendon matrix, dermal matrix, intestinal submucosa, pericardium matrix, and synthetic biomaterials are prepared. However, the current biological patches do not significantly promote enthesis regeneration. It has been reported that the energy-storing Achilles tendon shares common cellular and molecular inflammatory mechanisms with functionally distinct rotator cuff positional tendons^6^. Moreover, the three-dimensional structure and mechanical properties of Achilles tendon and rotator cuff are extremely similar^7^. Based on these characteristics, acellular porcine Achilles tendon (APAT) patch may provide sufficient mechanical support and matrix space for cell infiltration, proliferation, and differentiation in the early healing. And the APAT patch itself can also regulate cell activities and enhance cell infiltration and angiogenesis, thereby promoting rotator cuff repair^8^. However, the use of APAT patch may shield the host cells from mechanical signals required for optimal tissue remodeling during enthesis regeneration^9,10^. Thus, there is a demand for implantation of alternative stem cells for the application of acellular patches. Recently, researchers have tested the possibility of using tendon-derived stem cells (TDSCs) and the results have shown that the tendons contained more stem cells than the bone at the rotator cuff attachment site, indicating that the tendons might represent an important but neglected source of stem cells for postoperative enthesis regeneration^11^. Moreover, remarkable progress has been made with the identification of TDSCs^12,13^. TDSCs are characterized by multi-differentiation potential, including tenocytes, chondrocytes, osteocytes, and adipocytes. And apart from the multi-differentiation potential, TDSCs exhibit better osteogenic, chondrogenic and tenogenic in comparison with bone marrow stem cells under induction^14,15^. Meanwhile, inflammation does not seem to affect the proliferation rate of the isolated TDSCs and expression of differentiation marker genes^16^. These findings suggest that TDSCs might be a suitable cell source for enthesis regeneration approaches. In addition, TDSCs have been demonstrated to augment tendon healing and enthesis regeneration in several ways^17–19^.

The purpose of this study was to evaluate whether the composite of APAT patch and TDSCs could improve the histomorphological and biomechanical properties of the rotator cuff repair in a rabbit rotator cuff injury model. We hypothesized that utilization of the APAT patch encapsulating TDSCs as an underlay interposed between the supraspinatus tendon and the greater tuberosity of humerus would improve enthesis regeneration and collagen organization.

## Materials and methods

### Preparation and characterization of the APAT patch

All experimental protocols were approved by the Ethical Committee of Wuhan Fourth Hospital and performed in accordance with relevant guidelines and regulations. Achilles tendons were collected from a male adult pig subsequent to anesthesia with an intravenous injection of 3% pentobarbital sodium (30 mg/kg), followed by euthanization. The methodical excision of auxiliary tissues and enveloping fascia ensued. Storage at 4 °C in a solution of PBS containing 5% penicillin/streptomycin and 0.02% EDTA was implemented to counteract tissue degradation and inhibit bacterial proliferation. The specimens were subjected to a 12 h PBS lavage at 4 °C before use.

Referencing an earlier study^7^, the tendons were subjected to a treatment involving six chemical reagents to eradicate native cells: (i) 1% Triton-X 100 (Service Biotechnology Co., Wuhan, China); (ii) 1% sodium dodecyl sulfate (SDS, Service Biotechnology Co.); (iii) a custom-designed container containing ultrapure water supplemented with 100 U mL^−1^ penicillin/streptomycin (Gibco, Service Biotechnology Co.); (iv) 2% Triton X-100; (v) 3% SDS; and (vi) 100 U mL^−1^ DNase I (Service Biotechnology Co.). All processes were executed under aseptic conditions at the standard room temperature. Post-treatment, the samples underwent a comprehensive 24 h purification with ultrapure water, followed by a PBS rinse. Subsequently, the specimens were stored at 4 °C until use.

After decellularization, the tendon patches were examined under a JEOL TEM (Tokyo, Japan) with an accelerating voltage of 80.0 kV to confirm the complete removal of native cells and the preservation of collagen fiber continuity.

### Isolation and culture of rabbit TDSCs

Cell isolation method was based on previous studies^16,20^. Tendon-derived stem cells from 3 adult male rabbits were extracted using explant tissue granulation, fostering cell migration over a week. These cells were cultured in a basal medium containing high-glucose DMEM (Biossci, Wuhan, China), supplemented with penicillin/streptomycin/Fungiz-one and 10% fetal bovine serum. After the second passage, the cells were seeded onto the APAT patches (0.8 × 0.4 × 0.2 cm in dimension) at a density of 2 × 10^5^ cells per patch. A 2-day incubation period in basal medium preceded surgical implantation, facilitating cell attachment and growth on the patches.

### Adhesion and proliferation of TDSCs in the APAT patch

To visualize the adherent live TDSCs on the APAT patches, 4′-6-diamidino-2-phenylindole (DAPI) staining was used 24 h after cellular encapsulation. Population doubling time (PDT) was used to assess the proliferative capacity of these cells on the patches according to the previously published method^21^.

### In vitro differentiation experiment

To assess the tenogenic, chondrogenic, and osteogenic attributes of TDSCs, a comprehensive gene expression analysis was carried out utilizing quantitative polymerase chain reaction (qPCR). The second generation of stem cells were cultured in high-glucose DMEM, chondrogenic differentiation basal medium and osteogenic differentiation basal medium (Biossci) for 7 days and 14 days, respectively. The extraction of total RNA involved the utilization of the RNeasy Mini Kit with concurrent DNase I digestion (Biossci). Subsequent qPCR was conducted utilizing the QuantiTect SYBR Green PCR Kit (Biossci). Rabbit-specific primers were used to detect tenomodulin (Tnmd), scleraxis (Scx), aggrecan (ACAN), sex determining region Y box protein 9 (Sox9), alkaline phosphatase (ALP), and runt-related transcription factor 2 (Runx2), with the internal control being GAPDH (Table 1). The formulation of forward and reverse primers, as well as the size of resultant products, was guided by a previously established protocol^22^. To ensure robustness, a minimum of three independent experiments were conducted to determine the relative gene expression levels.

**Table 1:** Primer sequences of qPCR.

Gene	Forward 5′–3′	Reverse 5′–3′	Annealing temperature (°C)
Tnmd	AGAGTGGGCGAGG-TTCTGAA	CTGGCCGAGACAGC-TTCTGA	60
Scx	TGGAGCCCGAGGA-GAGGAGA	CCGCAGGTTTGAGG-TTTGGA	57
ACAN	AGTGTGAGGAGGC-GGTGTGA	AGTGTGAGGAGGCG-GTGTGA	57
Sox9	CCCGGAGGCGGAG-GAGGA	CTGTCCGTAGGTGGT-TGAGG	60
ALP	TGGAGAGTGAACG-CGTTCAC	CTCCAGGTCGCTTCA-CTTCC	58
Runx2	GGACGAGTTTTGC-CCAGGTT	GGCGTTTGGGTTGTT-GAGTT	58
GAPDH	TGAAGGTCGGAGT-CAACGGATTTGGT	CATGTGGGCCATGAG-GTCCACCAC	57

### Experimental animals and surgical procedure for the patch implantation

**Figure 1:**
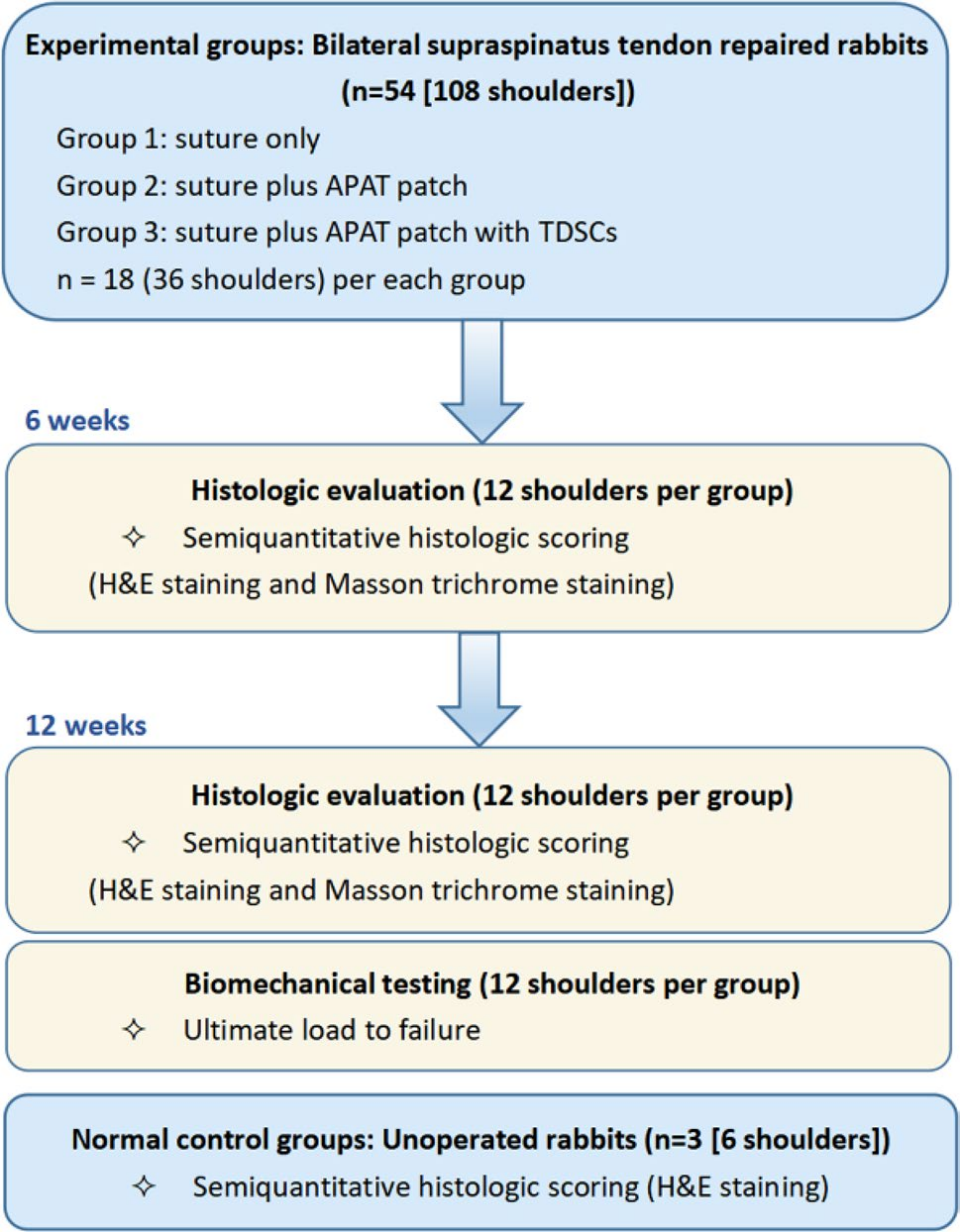
Allocation of experimental animals in this study. The intact tendon-to-bone sections (normal rabbits, n = 3) were set at 100% in semiquantitative histologic scoring system. *APAT patch* acellular porcine Achilles tendon patch, *TDSCs* tendon-derived stem cells.

**Figure 2:**
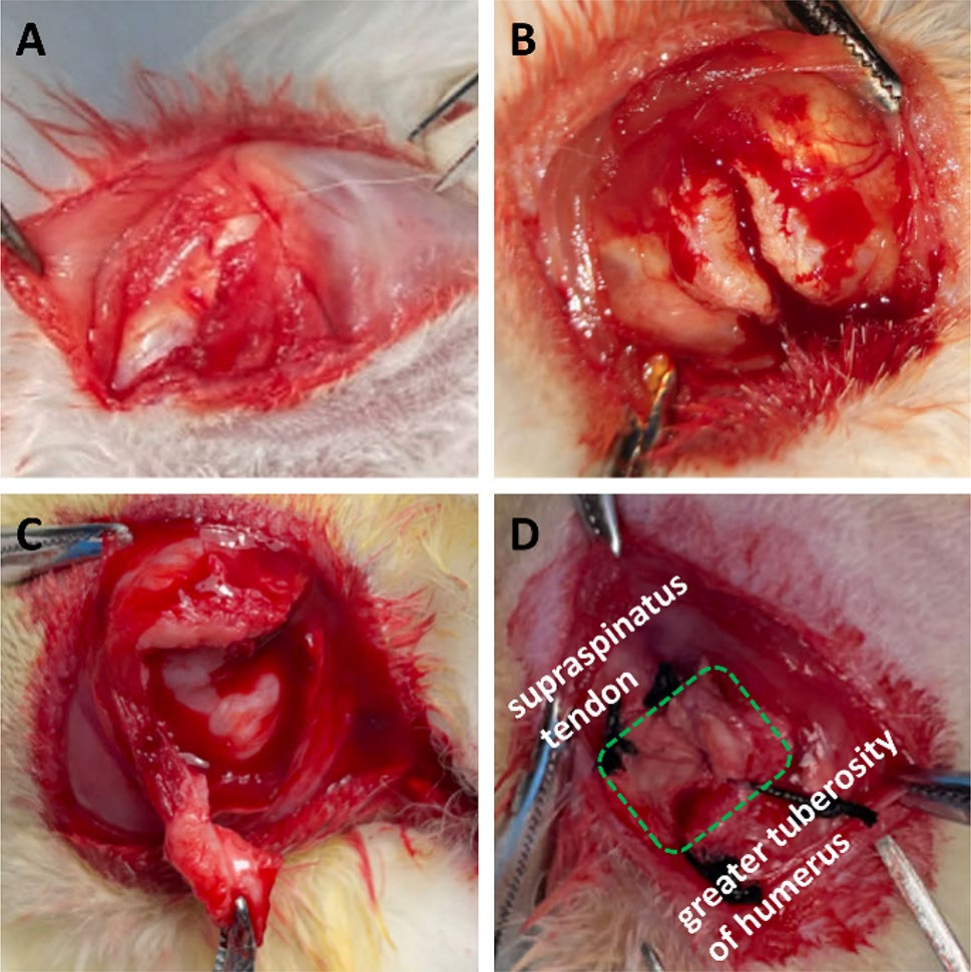
Schematic diagram of surgery. (**A**) The deltoid muscle was split to expose the supraspinatus tendon. (**B**) The rotator cuff was completely exposed, and the supraspinatus tendon at attachment site of the great tuberosity was transected. (**C**) Part of the distal supraspinatus tendon was removed with tissue scissors. (**D**) The APAT patch was sutured in a single row for repair in the original tissue position. The green area is the APAT patch.

All methods were reported in accordance with ARRIVE guidelines. A total of 57 adult male New Zealand White rabbits (6 months old with body weight of 3–3.3 kg) were received from the Animal Experiment Center of Wuhan Fourth Hospital. After 1-week acclimation, the rabbits were divided into distinct groups to perform bilateral surgical procedures. The rabbits were randomly assigned to one of the following groups: (1) control group (suture only, n = 18); (2) APAT patch group (suture plus APAT patch, n = 18); and APAT patch with TDSCs group (suture plus APAT patch with TDSCs, n = 18). The control group without APAT patch also underwent suture repair. Additionally, three extra animals were included as unoperated controls for histologic evaluation (Fig. 1). Animals were anesthetized with an intraperitoneal injection of 3% pentobarbital (40 mg/kg) and maintained at 3% isoflurane inhalation during surgery. With an external rotation of the arm, a 1.5-cm skin incision was made in an oblique sagittal position, and then the subcutaneous tissues were dissected layer by layer until the rotator cuff was exposed. The supraspinatus tendon was cut at the footprint with a scalpel and the fibrocartilage was removed. For patch implantation, the APAT patch was placed between the cut end of supraspinatus tendon and the footprint using a single row repair technique (Fig. 2). Intraperitoneal injection of Ceftezole sodium (2 ml, 40 mg/ml) was administered for three consecutive days after surgery to prevent infection. After surgery, the animals were allowed to move freely in their cages and they were sacrificed at 6 or 12 weeks post-surgery for histological analysis and biomechanical test.

### Histological analysis

Euthanasia was performed at 6 and 12 weeks postoperatively through overdose of pentobarbital sodium (50 mg/kg). The bilateral shoulders of 12 rabbits from each group were evaluated. After fixation and decalcification, specimens were bisected along the long axis of the tendon to expose the tendon-to-bone connection, and then paraffin-embedded to prepare 4-μm-thick sections. The tissue sections were stained with H&E to examine the morphologic characteristics, stained with α*-*SMA to examine the number of blood vessels, and stained with Masson trichrome to examine the fibrocartilage distribution. α-SMA was conducted utilizing the Strept Avidin–Biotin Complex kit (Biossci). α*-*SMA antibody (GB12045, Servicebio) served as the primary antibody in the α-SMA assay, while HRP-labeled goat anti-rabbit IgG (GB23303, Servicebio) was employed as the secondary antibody. Both antibodies were diluted at a ratio of 1:200. Masoon trichrome staining was performed using the Masson trichrome staining kit (Biossci). Samples were stained with prepared Weigert iron hematoxylin, differentiated with acid ethanol differentiation solution, and returned to blue with Masson blue solution. Then stained with Ponceau fuchsin staining solution, washed with weak acid solution, washed with phosphomolybdic acid solution, and stained with aniline blue staining solution. Finally, they were dehydrated and sealed with neutral gum. The sections were viewed under a Nikon Eclipse E100 microscope (Nikon Co., Tokyo, Japan), and digital images were taken using a Nikon DS-U3 camera for the microscope (Nikon Co.). Computerized image analysis was performed using Image J (NIH, Bethesda, MD) to evaluate the repair outcomes including cellularity, vascularity, and collagen fiber orientation in a semiquantitative manner. A modified histologic scoring system was used for each parameter (Table 2).

**Table 2:** Semiquantitative histologic scoring system.

Parameter	Scores			
	1	2	3	4
Cellularity, %*	> 400	300–400	200–300	< 200
Vascularity, bv/low PF	> 15	11–15	6–10	< 6
Collagen fiber orientation, %^†^	< 25	25–50	50–75	> 75

*bv* blood vessel, *PF* power field (low PF = 100-fold magnification).

*Enumeration of cells in designated regions of interest: each section underwent cell count quantification within specified regions of interest. The percentages delineated relative values compared to measurements from the baseline normal tendon-to-bone sections (n = 3), standardized at 100%.

^†^Measurement of grayscale in regions of interest: grayscale quantification within regions of interest was executed via Image J software across sections. The percentages conveyed intensity relative to measurements from reference normal tendon-to-bone sections (n = 3), with 100% serving as the calibration benchmark.

### Mechanical testing

**Figure 3:**
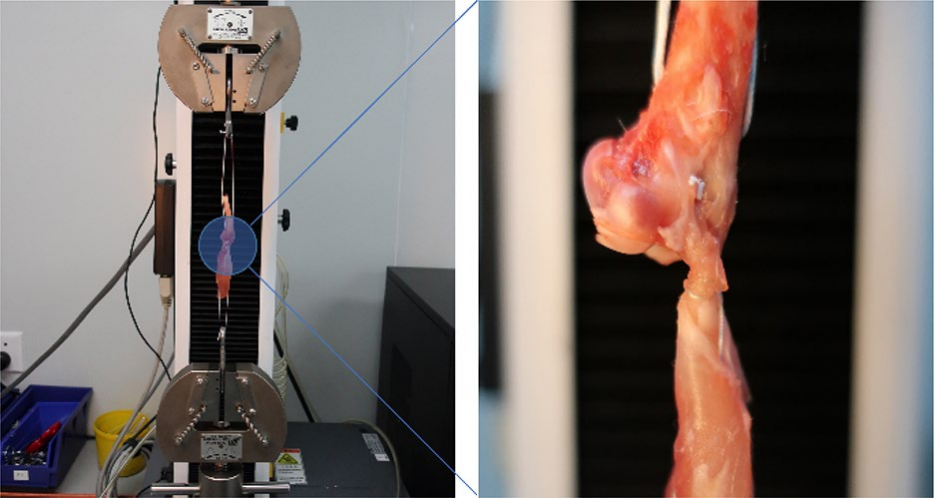
Uniaxial tensile test of the supraspinatus tendon-humerus unit. The supraspinatus tendon and humerus bone were hooked up on the two ends of the mechanical tester device.

Six rabbits from each group were tested for biomechanical properties 12 weeks after the surgery. All fresh specimens were frozen at − 80 °C until testing. After thawing, the grafted tendon to the humerus was dissected from the surrounding tissues and the supraspinatus muscle belly and any scar tissues were removed. Each specimen was preloaded to 10 N and then loaded to failure with a conventional tensile tester (STA1225; Orientec, Tokyo Japan) at a rate of 5 mm/s, and the ultimate load to failure and the failure site were recorded (Fig. 3).

### Statistical analysis

All statistical analyses of the data were performed with SPSS software version 26.0 (IBM, Chicago, USA). All data were presented as the mean ± standard deviation and all experiments were implemented at least three times. Student’s *t* tests (two-tailed unless otherwise stated) and one-way analysis of variance (ANOVA) were used to assess the statistical difference. *P < 0.05 was considered statistically significant.

## Results

### Removal of cellular components of the APAT patches

**Figure 4:**
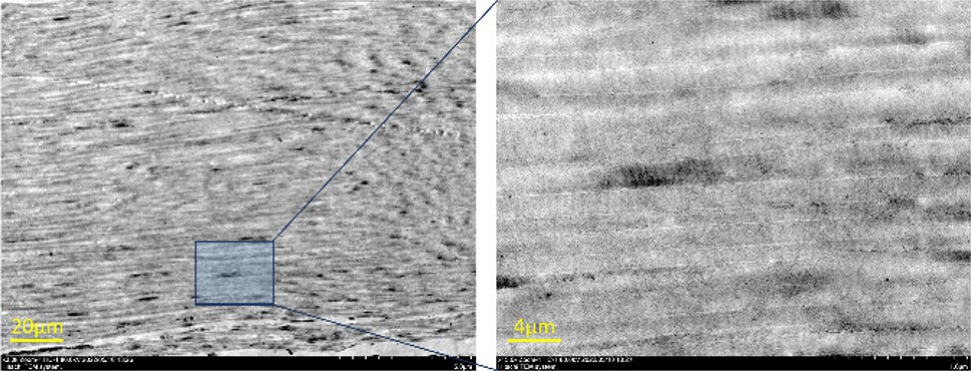
Microscopic images of the acellular porcine Achilles tendon (APAT) patch.

After decellularization, the patches appeared slightly swollen with no change in color. The results of TEM showed that the cells in situ in the patches were completely removed and the continuity of collagen fibers was maintained (Fig. 4).

### Adhesion and proliferation of TDSCs in the APAT patches

**Figure 5:**
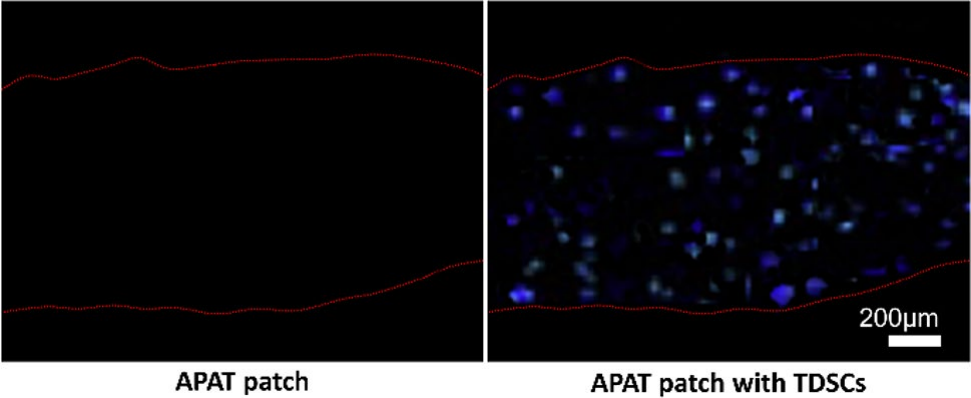
Adhesion of TDSCs in APAT patch. DAPI staining of APAT patch and APAT patch with TDSCs. The area within the red dashed line is the APAT patch. *APAT patch* acellular porcine Achilles tendon patch, *TDSCs* tendon-derived stem cells.

Twenty-four hours after cellular encapsulation, TDSCs were distributed homogeneously throughout the APAT patches (Fig. 5).

**Figure 6:**
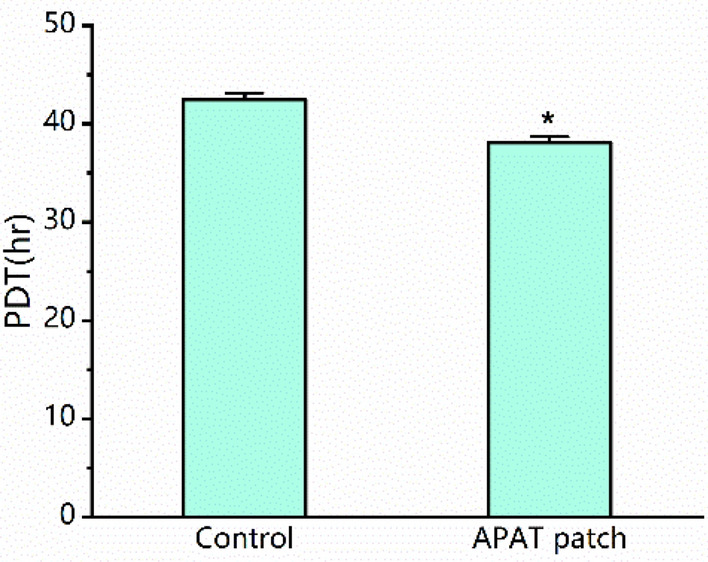
The population doubling time (PDT) of TDSCs on the APAT patches was significantly shorter than that of the same cells on the plastic surfaces (*P < 0.05). *APAT patch* acellular porcine Achilles tendon patch, *TDSCs* tendon-derived stem cells.

After 5 days in culture, TDSCs grew faster in the APAT patches than on the plastic surfaces, as shown by the population doubling times (PDTs) of TDSCs. The PDT of TDSCs in the APAT patches was about 10.4% shorter than that on the plastic surfaces (Fig. 6).

### Differentiation of TDSCs in the patches

**Figure 7:**
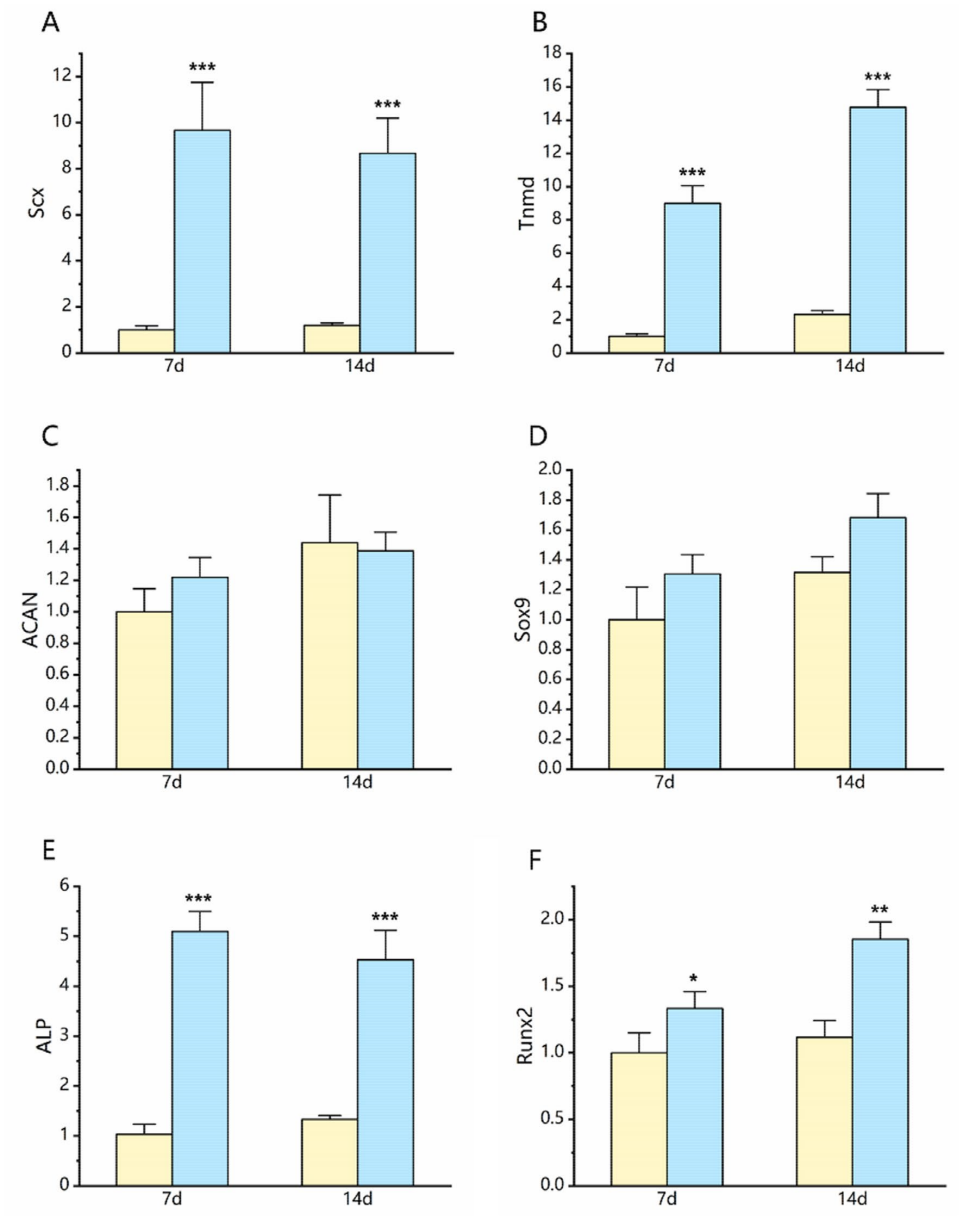
Multi-differentiation potentials of TDSCs in the APAT patches and on the plastic surfaces (the control group). After being cultured in the respective induction media for 7 days and 14 days, expression of tenocyte-related and osteocyte-related genes in the APAT patches was significantly higher than those cells cultured on the plastic surfaces. (**A**,**B**) Expression of Scx and Tenomodulin. (**C**,**D**) Expression of ACAN and Sox9. (**E**,**F**) Expression of ALP and Runx2. *P < 0.05, **P < 0.1, and ***P < 0.001. Of note, gene expression levels were normalized using the values in the corresponding control groups. Yellow bars, the control group; blue bars, the APAT patch group.

We examined the multi-differentiation potentials of TDSCs by culturing them in tenogenic, chondrogenic, and osteogenic induction media, respectively. Three sets of genes included tenocyte-related genes (Scx and Tnmd), chondrocyte-related genes (Sox9 and ACAN), and osteocyte-related genes (ALP, Runx2). We found that tenocyte-related and osteocyte-related genes were significantly up-regulated in the TDSCs cultured in the APAT patches compared with that in the control group cultured in the plastic dishes. Expression of chondrocyte-related genes (Sox9 and ACAN) in the APAT patches was not notably higher than those in the control group. These results suggest that the APAT patches markedly promote TDSCs differentiation to tenocytes and osteocytes (Fig. 7).

### H&E staining

Six weeks after surgical repair, fresh granulation tissues with highly vascularized and disordered arrangement was observed in the control group. In the APAT patch group and APAT patch with TDSCs group, the formation of dense and orderly collagen fibers was observed. Moreover, the collagen fiber density of the APAT patch with TDSCs group was higher than that of the APAT patch group. However, there was not any typical three-layered structure in any group.

**Figure 8:**
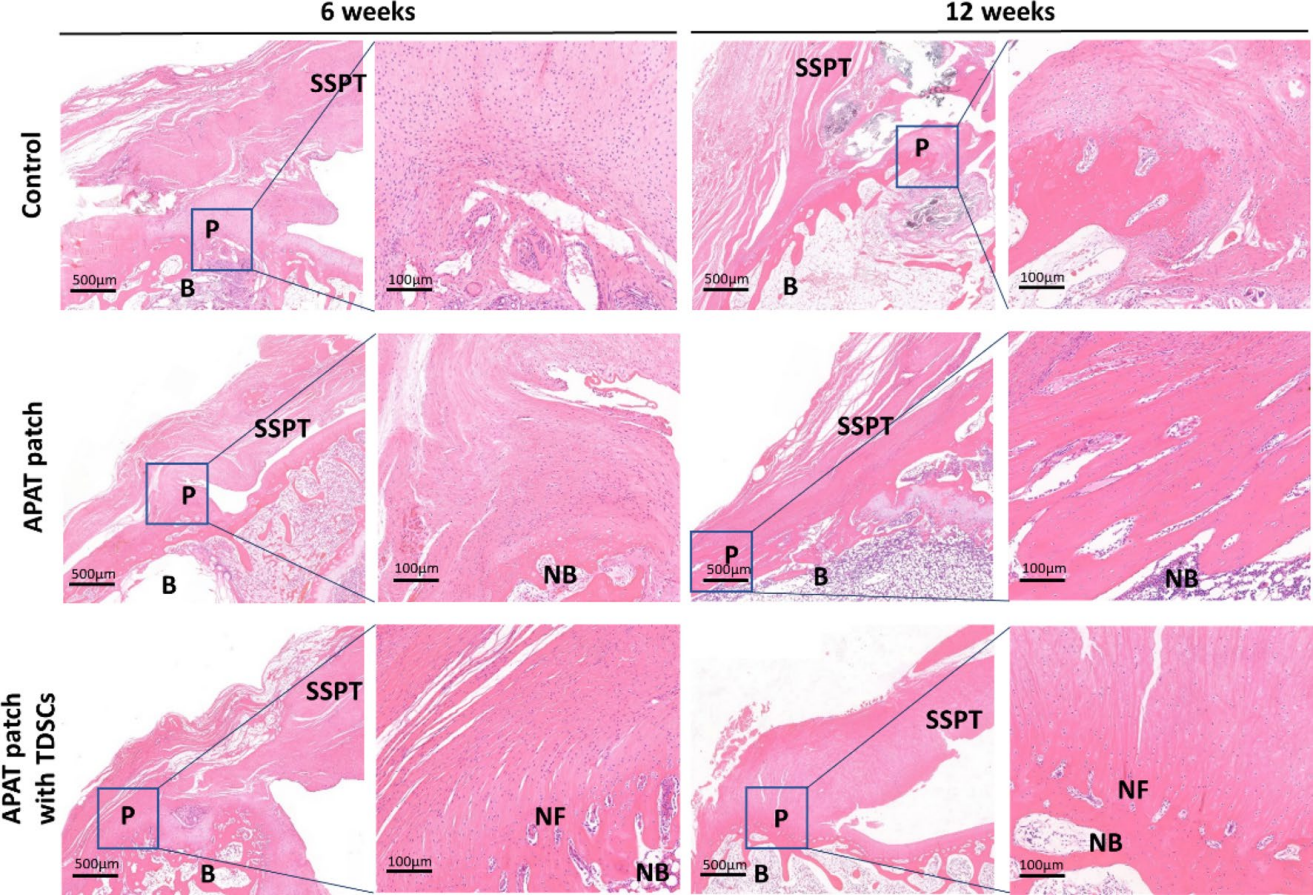
Photomicrographs of the tendon-to-bone interface in the 3 groups, taken at 6 and 12 weeks after surgery. The tissue sections were stained with H&E staining. The boxed areas in the left panels from each group at each time point are shown at a higher magnification in the right panels. *APAT patch* acellular porcine Achilles tendon patch, *TDSCs* tendon-derived stem cells, *B* bone, *P* patch, *SSPT* supraspinatus tendon, *NB* new bone, *NF* new fibrocartilage.

Twelve weeks after surgical repair, dense and disordered collagen fibers were observed in the control group. In the APAT patch group and APAT patch with TDSCs group, the formation of collagen fibers was denser and the improvement was more obvious that at six weeks. Moreover, an obvious three-layered structure was observed in the APAT patch with TDSCs group (Fig. 8).

### Masson trichrome staining

Six weeks after surgical repair, there was no fibrocartilage and bone formation at the tendon-to-bone interface in the control group. A little bone formation was observed in the APAT patch group. A little fibrocartilage and bone formation were observed in the APAT patch with TDSCs group, which were closely continuous with the tendon tissue.

**Figure 9:**
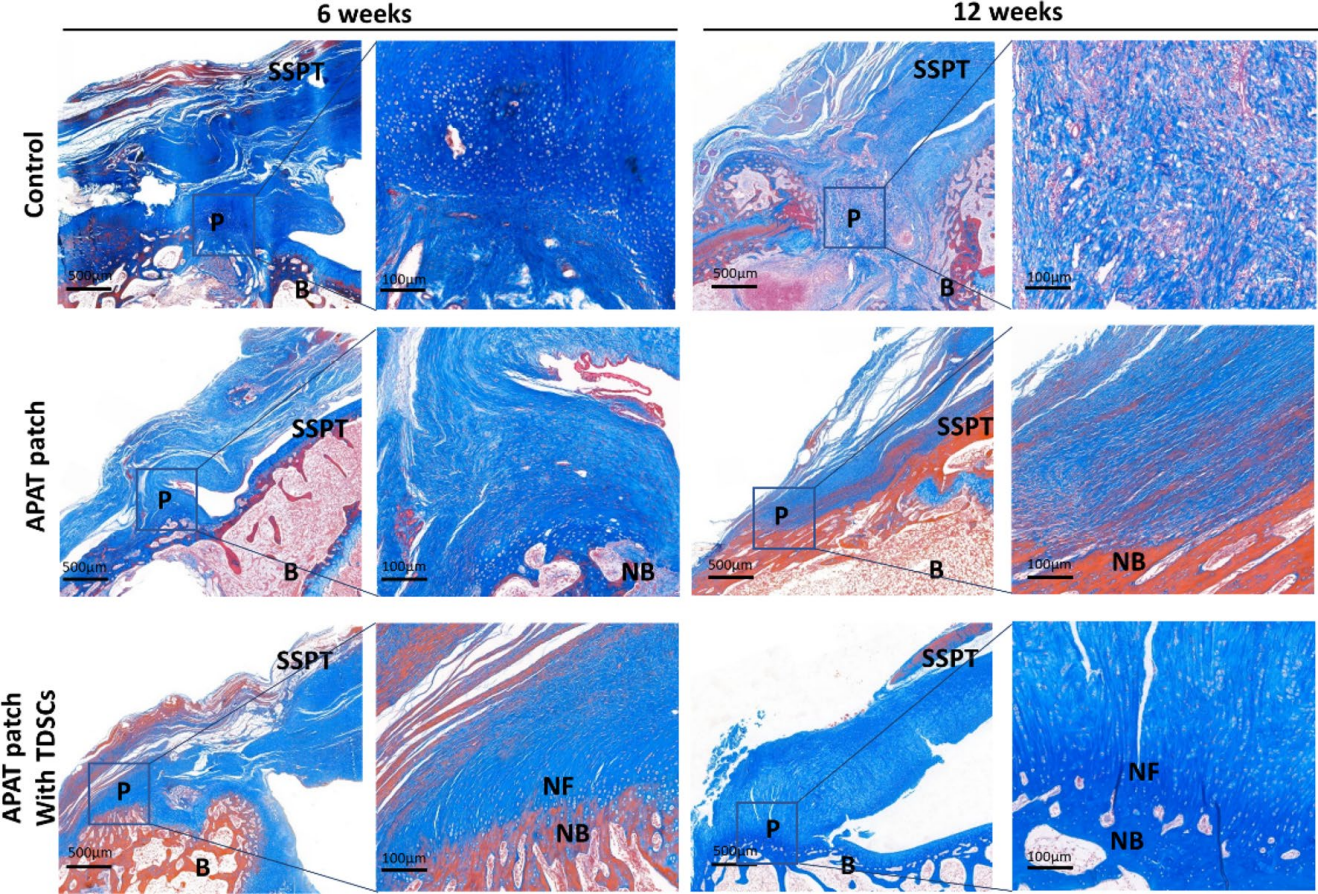
Photomicrographs of the tendon-to-bone interface in the 3 groups, taken at 6 and 12 weeks after surgery. Tissue sections were stained with Masson trichrome staining. The boxed areas in the left panels from each group at each time point are shown at a higher magnification in the right panels. The red areas in the supraspinatus tendon and patch are muscle fiber tissue, the red areas of bone tissue are mature bone tissue, the red and blue mixed areas are new bone tissue, and the blue areas are collagen fibers. *APAT patch* acellular porcine Achilles tendon patch, *TDSCs* tendon-derived stem cells, *B* bone, *P* patch, *SSPT* supraspinatus tendon, *NB* new bone, *NF* new fibrocartilage.

Twelve weeks after surgical repair, fibrocartilage and bone formation were still absent in the control group. The collagen distribution was denser and more orderly compared with that at 6 weeks in the APAT patch group. The number of fibrocartilage and bone in the APAT patch with TDSCs group was significantly increased compared with that at 6 weeks. In addition, more bone tissue was observed in the APAT patch with TDSCs group than in the APAT patch group (Fig. 9).

### α-SMA staining

**Figure 10:**
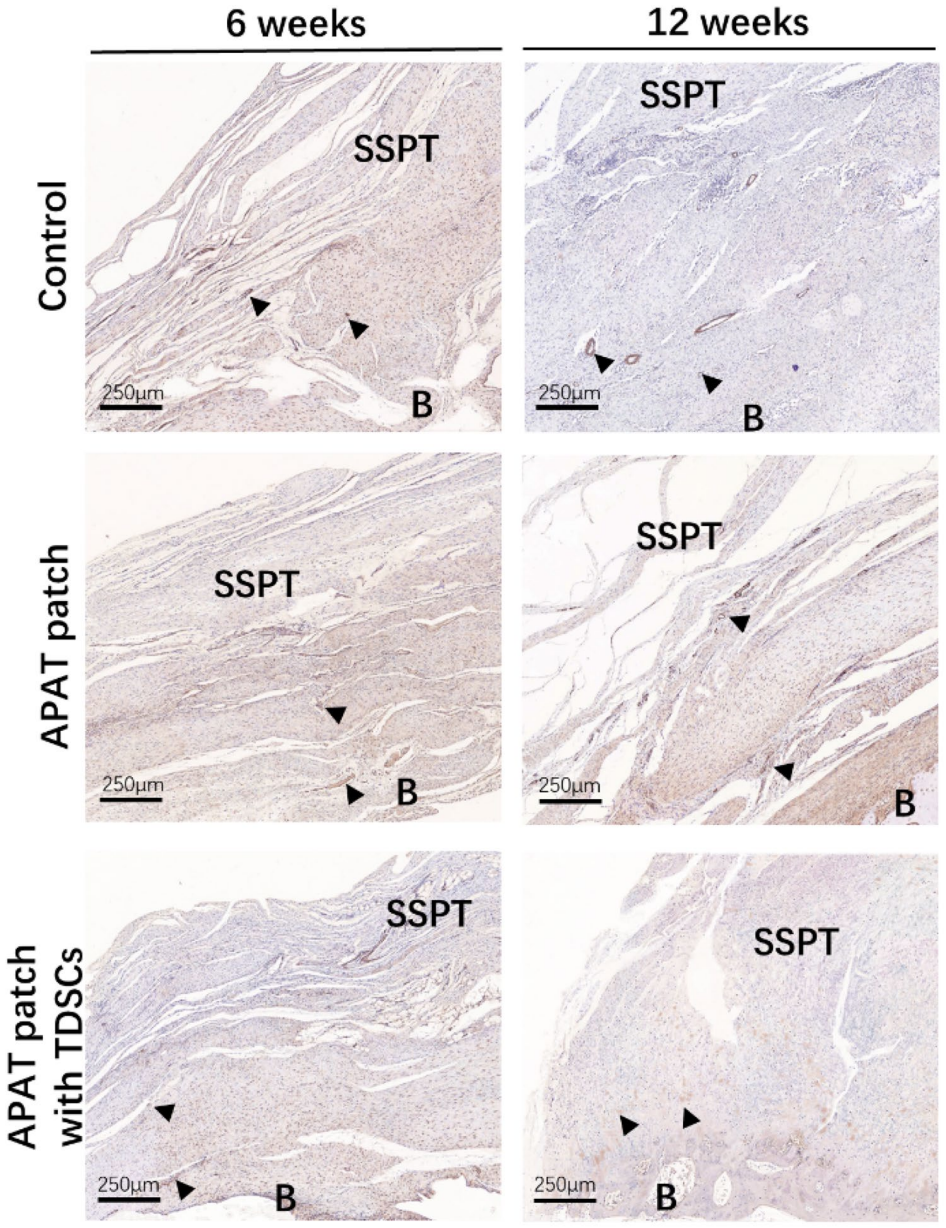
Photomicrographs of the tendon-to-bone interface in the 3 groups, taken at 6 and 12 weeks after surgery. Tissue sections were stained with α-SMA. The arrows indicate the blood vessels. *APAT patch* acellular porcine Achilles tendon patch, *TDSCs* tendon-derived stem cells, *B* bone, *P* patch, *SSPT* supraspinatus tendon.

At 6 and 12 weeks, the number of blood vessels in the APAT patch with TDSCs group was significantly less than that in the control and APAT patch groups (Fig. 10).

### Histologic evaluation

Twelve shoulders from each group were evaluated at 6 and 12 weeks postoperatively. The results of semiquantitative histologic evaluation are presented in Table 3.

**Table 3:** Results of histologic evaluation.

Groups	Cellularity, %*	Vascularity, bv/low PF^✝^	Collagen fiber orientation, %^‡^	Total score^§^
At 6 weeks				
Group 1	353 ± 46.7	18.2 ± 5.3	50.2 ± 5.9	5.4 ± 1.1
Group 2	413 ± 44.9	10.2 ± 5.1	57.5 ± 5.1	6.5 ± 0.9
Group 3	479 ± 55.5^#-‖^	8.7 ± 3.8^#^	78.2 ± 6.4^#-‖^	9.2 ± 0.9^#-‖^
At 12 weeks				
Group 1	202 ± 46.4	14.3 ± 4.6	61.5 ± 8.6	8.0 ± 1.1
Group 2	219 ± 47.3	8.9 ± 3.4	73.3 ± 8.9	9.4 ± 1.3
Group 3	289 ± 44.8^#-‖^	7.3 ± 3.1^#^	87.1 ± 8.2^#-‖^	12.5 ± 1.6^#-‖^

n = 6 for all groups. Values are expressed as the mean ± standard deviation. Group 1 = suture only; group 2 = suture plus APAT patch; group 3 = suture plus APAT patch with TDSCs.

*bv* blood vessel, *APAT patch* acellular porcine Achilles tendon patch, *TDSCs* tendon-derived stem cells.

*Number of cells per region of interest from each section. The percentages represent relative values compared with the values from the normal tendon-to-bone sections (1320 ± 159 cells/mm^2^, n = 3), which were set to 100%.

^✝^Number of blood vessels per low power field (100× magnification) from each section.

^‡^Grayscale per region of interest from each section as measured using Image J. The percentages represent relative values compared with the values from the normal tendon-to-bone sections (139.75 ± 14.80 grayscale, n = 3), which were set to 100%.

^§^Scores represent a total score of three parameters, including cellularity, vascularity, and collagen fiber orientation.

^#-‖^Significantly different between groups compared with group 1 (^#^) and group 2 (^‖^) at the same time point (*P* < 0.05).

The cellularity decreased significantly over time in all groups (*P* < 0.001). At 6 weeks and 12 weeks, the cellularity was significantly higher in the group 3 (*P* < 0.001 vs. Group 1, *P* < 0.05 vs. Group 2).

The number of blood vessels was significantly different with respect to the time points in all groups (*P* < 0.05). In group 3, it decreased significantly compared with group 1 (*P* < 0.05), although not with group 2 (*P* > 0.05) at 6 weeks. Similarly, the number of blood vessels of group 3 decreased significantly compared to group 1 (*P* < 0.05), although not with group 2 (*P* > 0.05) at 12 weeks.

The value of collagen fiber orientation significantly increased over time in all groups (*P* < 0.05). There were significant differences between the groups at 6 weeks (group 3 vs Group 1, *P* < 0.001; group 3 vs Group 2, *P* < 0.001). There were significant differences between the groups at 12 weeks (group 3 vs Group 1, *P* < 0.05; group 3 vs Group 2, *P* < 0.05).

The total histologic score was significantly increased over time in all groups (*P* < 0.001). At 6 weeks, the total score was significantly higher in group 3 than in other two groups (*P* < 0.001). And similarly, it was significantly different between the groups at 12 weeks (group 3 vs Group 1, *P* < 0.001; group 3 vs Group 2, *P* < 0.05).

### Biomechanical testing

**Figure 11:**
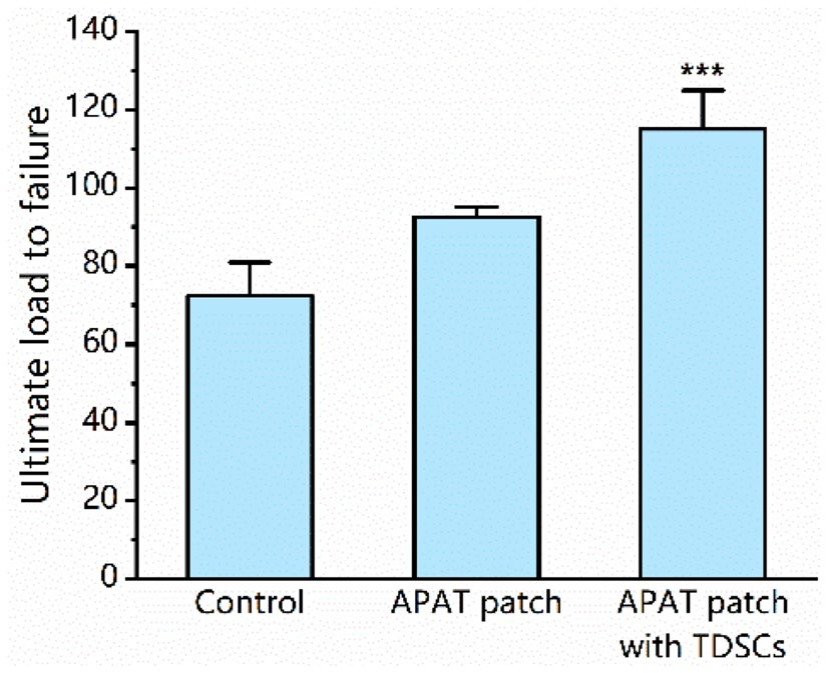
Tensile loading results. Changes in mechanical strengths were evaluated as the ultimate loads to failure. ***P < 0.001 compared to other two groups. *APAT patch* acellular porcine Achilles tendon patch, *TDSCs* tendon-derived stem cells.

We tested 36 specimens (12 shoulders from each group) from the supraspinatus tendon-to-bone repair constructs at 12 weeks postoperatively. All experimental specimens failed at the repair site during testing. At 12 weeks, the ultimate load to failure was 72.32 ± 8.54 N in the control group, 92.58 ± 2.57 N in the APAT patch group, and 115.06 ± 19.36 N in the APAT patch with TDSCs group (Fig. 11). The ultimate load to failure was significantly higher in the APAT patch with TDSCs group than in other two groups (*P* < 0.001).

## Discussion

In this study, acellular porcine Achilles tendon (APAT) patch was combined with tendon-derived stem cells (TDSCs) to promote rotator cuff tendon-to-bone healing. Acellular Achilles tendon tissue could provide static structural stability and dynamic biomechanical adaptability. TDSCs could differentiate into different cells to fill the rotator cuff tendon-to-bone interface. The results of in vitro experiments showed that expression of tenogenic and osteogenic genes was increased from 7 to 14 days after the TDSCs was seeded on the APAT patch. In addition, histological results showed that the APAT patch with TDSCs significantly promoted tendon and bone tissue regeneration and enhanced tendon-to-bone healing.

Biological patch technology has been widely used in the treatment of rotator cuff tears and can provide good mechanical support in the early stage of repair. The biological patch can change the local biomechanical environment and reduce the stress in the rest of the rotator cuff^23^. Generally, patches from allogenic origin can provide better results. However, patches also are associated with a higher cost and the need for tissue donor availability in clinical practice, and the clinical effect of biological patch was not good and the incidence of re-tear was high. Therefore, biological patches from xenogenic origin are more of interest to study. Patrick et al.^24^ conducted a multicenter study of acellular dermal to repair massive rotator cuff tears, and the results showed that the success rate was less than 70% after one year. The authors speculated that the reason might be that the biological patch could only provide biomechanical reinforcement in the early stage and could not regenerate the multi-layered structure of the tendon-to-bone interface at the histological level. Several Studies have shown that Achilles tendon and rotator cuff share common cellular and molecular inflammatory mechanisms, as well as a very similar three-dimensional structure^6,7^. However, tissues with fewer endogenous cells lack the intrinsic ability to restore their full mechanical strength and function, and acellular matrix alone may not be sufficient to achieve tendon-to-bone interface regeneration^25^. TDSCs have been shown to be very effective stem cells for tissue repair, as they can be directed to differentiate into tenocytes, chondrocytes, and osteocytes^16^. TDSCs have also been shown to play a role in filling the tendon-to-bone interface in animal models. While the acellular matrix has great biological properties, TDSCs have an excellent biological enhancement effect in the tendon-to-bone healing. However, there is no any in vitro or in vivo study on the application of acellular Achilles tendon patch combined with tendon stem cells for rotator cuff tendon-to-bone healing.

In order to study the application of the APAT patch combined with TDSCs in rotator cuff tendon-to-bone healing, we prepared the APAT patches according to a previous differential acellular treatment^26^. Regarding the sterile treatment of APAT patch, we have considered many ways, including alcohol immersion, high temperature disinfection, ultraviolet irradiation, and addition of antibiotics. However, all these methods significantly changed the physical properties of the patch except the addition of antibiotics. By adding penicillin/streptomycin, the physical properties and chemical structure of the patch could be preserved as much as possible while being treated aseptically. However, we hypothesized that this approach would still affect the biological activity of the matrix and the ability to support cell migration, proliferation and differentiation, and the biomechanical properties would be reduced compared with normal Achilles tendon tissue. Generally, patches from allogenic origin can provide better results. In addition, we established a rotator cuff injury model and used the APAT patch encapsulated with TDSCs for rotator cuff repair. Histological results showed that the number of vertical collagen fibers at the attachment site increased and a small amount of bone tissue regeneration could be seen after APAT patch and APAT patch with TDSCs were used for rotator cuff repair. With the passage of time, the collagen fibers at the attachment point became denser and more orderly, and gradually grew inward, mineralized and fused. In addition, in the APAT patch with TDSCs group, we observed a continuous and clear three-layered gradient structure. However, there was no obvious three-layered gradient structure in the APAT patch group. This may be related to the degradability of acellular matrix in vivo. A large number of studies have shown that acellular matrix is usually completely degraded within 28 to 90 days after implantation, resulting in a gradual decrease in the activity and biomechanical strength provided by the matrix^27–30^. In addition to improving local tissue stress, acellular matrix is also related to cell proliferation and differentiation and in situ cell growth^8^. Therefore, while the biomechanical strength of the APAT patch is decreased, TDSCs can proliferate and differentiate, instead of degrading the collagen matrix. In addition, after implantation of acellular matrix, cell activity was closely regulated, cell infiltration and vascularization were enhanced^27^. Therefore, denser and more ordered fibrous collagen and continuous and clear three layers of gradient structure could be observed in the APAT patch with TDSCs group. In addition, no obvious fibrocartilage formation was observed in the APAT patch group at 6 and 12 weeks. The results of qPCR analysis showed that gene expression levels of chondroblasts did not increase significantly within 7 to 14 days. ACAN expression and extracellular matrix play an important role in the differentiation and maturation of chondroblasts^31^. Sox9 plays a key role in chondrocyte differentiation, and its expression is associated with multiple stages of chondrocyte differentiation^32^. This result is consistent with the conclusion of a previous study. Ide et al.^33^ used acellular dermal matrix patch to repair rotator cuff defect and the results showed that the number of chondrocytes, the percentage of chondrocytes aligned in rows, and the area of the fibrocartilage layer were significantly smaller than in the control group. At 6 and 12 weeks, a small amount of fibrocartilage formation was observed in the APAT patch with TDSCs group. It may be related to the differentiation of TDSCs into chondrocytes in vivo. However, the expression levels of chondrocyte markers in the APAT patch group were similar to those in the plastic group at 7 and 14 days. This result indicated that APAT patch had started to promote the differentiation of TDSCs into chondrocytes at 6 weeks, but there was no significant difference in the short term. In addition, obvious tendon tissue was observed in both the APAT patch with TDSCs group and APAT patch group. The qPCR analysis showed that the gene expression levels of tenocytes and osteocytes were increased significantly within 7–14 days. ALP is an early marker of osteoblast differentiation and maturation, especially after the transformation from preosteocyte to osteocyte^34^. Runx2 is expressed in the early stage of healing and plays an important role in the differentiation and maturation of osteoblasts^35^. Scx is expressed in all stages of tendon development, which plays an important role in tendon genesis, differentiation and regeneration, and it is a relative specific molecular marker of tendon^36^. Tnmd is a specific molecular marker of the tendon and plays an important role in the development and maturation of tendon^37^. This result is consistent with the histological observation, indicating that the APAT patch can promote the differentiation of TDSCs into tendon cells and osteocytes and enhance the rotator cuff tendon-to-bone healing. The results of histological semi-quantitative evaluation showed that the cell proliferation was more obvious and the collagen fibers were more orderly in the APAT patch with TDSCs group. In addition, the biomechanical test results showed that the ultimate load of the APAT patch with TDSCs group was significantly higher than that of the APAT patch group and control group. The results showed that compared with the APAT patch alone, the APAT patch implanted with TDSCs could significantly improve the biomechanical strength between tendon and bone.

This study has some limitations. First, we did not establish a chronic rotator cuff injury model, as most of the clinical patients with giant rotator cuff tears are slow degenerative tears, but our model cannot reflect the healing process of patients. Secondly, we have not studied the role of TDSCs alone in rotator cuff tendon bone healing, but a large number of studies have shown that TDSCs play an active role in rotator cuff tendon bone healing^13,38,39^. Finally, our study reflects the early process of rotator cuff tendon bone healing and does not follow up the medium-and long-term effects of the patch materials.

To sum up, the APAT patch implanted with TDSCs can significantly promote the repair of rotator cuff healing and enhance the biomechanical strength of tendon interface. In the end, these results may provide new ideas for the study of biological patches in rotator cuff tendon bone healing and hopefully provide a new method for clinical treatment of rotator cuff tears.

## Conclusion

This study is the first to combine acellular porcine Achilles tendon patch with tendon stem cells for rotator cuff repair. In vitro studies have shown that acellular porcine Achilles tendon patch can significantly improve the differentiation ability of tendon stem cells, especially into tenocytes and osteocytes. In addition, the APAT patch encapsulated with TDSCS could significantly promote tendon-bone healing of rotator cuff in vivo. Therefore, we believe that the combination of the APAT and stem cells is an effective method for the treatment of rotator cuff injury.

## Data Availability

The datasets used and/or analysed during the current study available from the corresponding author on reasonable request.
